# Trace level detection of Pb^2+^ ion using organic ligand as fluorescent-on probes in aqueous media

**DOI:** 10.1016/j.heliyon.2024.e41125

**Published:** 2024-12-12

**Authors:** Maria Sadia, Jehangir Khan, Rizwan Khan, Syed Wadood Ali Shah, Adil Zada, Muhammad Zahoor, Riaz Ullah, Essam A. Ali

**Affiliations:** a Department of Chemistry, University of Malakand, Chakdara, Lower Dir, Khyber Pakhtunkhwa, Pakistan; b Department of Electrical Engineering, Kwangwoon University, Seoul, South Korea; c Department of Pharmacy, University of Malakand, Chakdara, Lower Dir, Khyber Pakhtunkhwa, Pakistan; d Department of Biochemistry, University of Malakand, Chakdara, Lower Dir, Khyber Pakhtunkhwa, Pakistan; e Department of Pharmacognosy, College of Pharmacy, King Saud University, Riyadh, Saudi Arabia; f Department of Pharmaceutical Chemistry, College of Pharmacy King Saud University, Riyadh Saudi Arabia

**Keywords:** Sensitivity, Optical probe, Toxic metals, Aqueous solution, DFT studies

## Abstract

In this study, an optical sensor, JA/(2,6-di((E)-benzylidene)cyclohexan-1-one), was synthesized and characterized using ^1^H NMR and FT-IR spectroscopy. The sensor exhibited high efficiency and selectivity in detecting Pb^2+^ ions, even in the presence of potential interfering ions such as Mn^2+^, Cu^2+^, Co^2+^, Cr^3+^, Ni^2+^, Ce^3+^, Hg^2+^, and Cd^2+^ in aqueous solutions. The interaction of JA with Pb^2+^ resulted in a significant enhancement of fluorescence intensity, suggesting the formation of a stable complex. A 2:1 binding ratio between JA and Pb^2+^ was confirmed through fluorometric analysis, absorption spectra, and theoretical calculations (DFT). The association constant for the complex was calculated to be 3 × 10^6^ M^−2^. The sensor demonstrated high sensitivity towards Pb^2+^ with a detection limit of 5 × 10^−7^ M. Additionally, JA was successfully reused by applying EDTA to release Pb^2+^ from the sensor. Real sample analysis under optimized conditions of pH, time, and concentration of JA and Pb^2+^ further validated the practical applicability of the sensor.

## Graphical abstract

**Figure:**
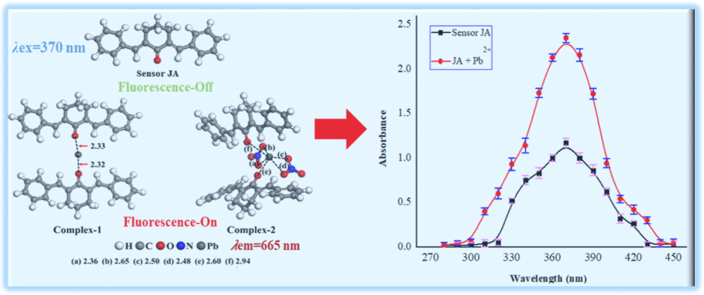

## 1 Introduction

Due to the increasing expansion of industrialization, the problem of water contamination has become more serious globally [1]. Ecological degradation is the biggest obstacle facing present human society [2]. Heavy metal pollution and ecological deterioration brought on by their presence in the environment have become a serious and worrying problem [3]. Consistently thrown into aquatic systems include both home and industrial garbage, frequently including significant levels of heavy metal ions [4]. Heavy metal ions demonstrate high toxicity due to their capability to coordinate, which has a direct or indirect effect on the environment and human health. Even at low quantities, lead (Pb^2+)^ ion stands out among these heavy metal ions as being particularly dangerous for both human health and marine life [5]. In addition, the excessive accumulation of Pb^2+^ in the human body will not only seriously damage the central nervous system, but also induce diseases such as Alzheimer's disease. Pb^2+^ salts that are soluble or barely water-soluble can easily enter biological systems through polluted water and soil, having a significant negative effect on the lives of organisms. According to Wani et al., in 2015, this incursion seriously hampers crucial plant activities such as photosynthesis, water management, nitrogen metabolism, and cell division [6]. Various toxicological manifestations of Pb^2+^ exposure's adverse effects in humans have been thoroughly reported [7]. Given the harmful effects of Pb^2+^, the action level established by the EPA in 1996 is 15 μg/L, which was also acknowledged by WHO in 2011. The acceptable threshold for drinking water is set at 10 μg/L [8]. However, these established limitations are frequently exceeded globally due to the presence of Pb^2+^ in both groundwater and surface water. As a result, it becomes crucial to remove Pb^2+^ from water sources to protect the public's health. Several methods have been developed to remove Pb^2+^ ions from aqueous samples. These include reverse osmosis [9], solid-phase extraction [10], co-precipitation [11], liquid-liquid extraction [12], nano-filtration [13], ion exchange, flotation, adsorption, amperometric analysis and electrochemical deposition [14,15]. These approaches have drawbacks because of their high operational costs, difficulty of mobility of the instruments, complex sample preparation, and demand for specialist technical expertise despite their impressive sensitivity and selectivity. Therefore, there is an immediate demand for the emergence of simple, quick, considerate, and economical methods. Due to its easiness, cheapness, fast interaction, and environmental friendliness, fluorescence-based detection of Pb^2+^ ions has garnered significant interest from researchers [16,17]. Fluorescence-based sensors do, however, have certain limitations, including poor selectivity, low probe solubility, and significant susceptibility to interference from other metals [[18], [19], [20]]. Fluorescent sensors based on organic probes have been recorded, providing enhanced and more efficient fluorescence-based Pb^2+^ detection, to overcome these constraints [21,22]. These organic sensors having good optical properties, significant solubility, and stability enabling them to detect Pb^2+^ with efficient fluorescence response [[23], [24]].

Curcumin derivatives are widely used in different disciplines including medicine, nanomaterials and gastronomy. These derivatives possess good optical properties. Optical properties of curcumin derivatives are owing to the existence of pH, additives and polarity of solvent. Due to the easy synthesis and high sensitivity in solvent environment, they are widely used for metal ion detection. Curcumin exists in two forms keto and enol, the enol form allows the molecule to accept and donate electrons, and further due to the availability of enol form it can act as metal chelator for metal ions. Curcumin plays a key role in electrochemical transducer and ion receptor. It is also capable for metal ion detection in its tautomeric form in the course chelation, thereby exhibiting cathodic and anodic shift. Curcumin based sensors based on fluorescence show elevated sensitivity, reproducibility and small detection limit. Additionally, by minimizing Intramolecular Charge Transfer (ICT) occurrences, these probes are especially good at building compounds with divalent metal ions like Pb^2+ 24^. These metal ion complexes also increase optical responses at specific excitation and emission wavelengths. The signals resulting from changes in fluorophores' light emission and absorption provide information on the process behind the detection of Pb^2+^ ions. Niu et al. [25], Wang et al. [26], Venkateswarlu et al. [27], Wang et al. [28], and Aggarwal et al. [29], Ghorai et al. [30], Sharma et al. [31], have designed and synthesized fluorescent sensors based on organic moiety for the detection of Pb^2+^. However, these organic fluorescence-based sensors still have inadequate sensitivity and selectivity. Thus, the development of optical sensors for divalent ions like Pb^2+^ in aqueous media is still challenging and very attractive. Therefore, the focus of this work was on developing a fluorescence-on sensor based on a curcumin derivative (designated as sensor **JA**). This derivative was developed to provide an enhanced selectivity and sensitivity for detecting Pb^2+^ ions in aqueous environments with apparent enhancement in fluorescence intensity. Because of its carefully selected coordination sites, the sensor **JA** demonstrated the ability to form long-lasting complexes with Pb^2+^ ions. The probe capacity to preferentially link with Pb^2+^ ions in the presence of several other metal ions served as its fundamental operating principle.

## 2 Experimental

### 2.1 Chemicals and reagents

**Fig. 1:**
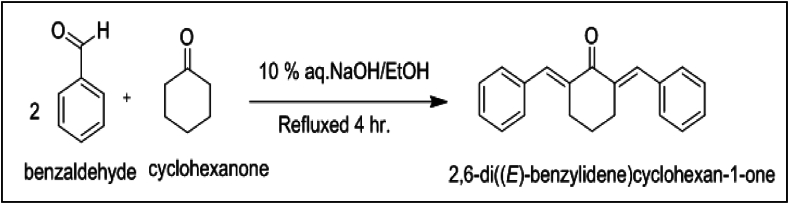
Chemical structure of optical sensor **JA** (2,6-di(*E*)-benzylidene) cyclohexane-1-one).

The materials and reagents used in the current research were high-grade purity and used without any further processing; the water was double distilled before use. The salts of Mn(OH_3_), Co(OH_3_), NiSO_4_, Cd(SO_4_)_2_·3H_2_O, Hg(NO_3_)_2,_ Cu(SO_4_)_2_·5H_2_O, CrCl_3_, Pb(NO_3_)_2,_ Al_2_(SO_4_)_3_, BiCl_3_, CrSO_4_, Fe SO_4,_ BaCl_2_, KCl, Na_2_CO_3_, Zncl, CrSO_4_, AS_2_O_3_, and Ce(NO_3_)_3_ metals were obtained from BDH chemical, England. Sodium hydroxide, cyclohexanone (ketone), benzaldehyde (aldehyde), methanol, and acetonitrile were obtained from Sigma-Aldrich. UV–Vis spectra were recorded using a double-beam UV/VIS spectrophotometer (Shimadzu, Japan, model 1601). Optical analysis was conducted with a spectrofluorometer (model RF5301 PC, Shimadzu, Japan). Infrared (IR) spectra and proton nuclear magnetic resonance (^1^H NMR) spectra were obtained using an FT-IR spectrophotometer (model 1601, Shimadzu, Japan) and a Bruker Advance 400 MHz spectrometer (Prestige 21, Shimadzu, Japan), respectively. The ^1^H NMR spectra were recorded in CDCl₃ solution, using tetramethylsilane (TMS) as an internal standard. The structure of the synthesized sensor JA is shown in Fig. 1.

### 2.2 Preparation of solutions for spectroscopic measurements and solvent used

For the spectrophotometric analysis, a stock solution of the optical sensor JA was prepared in acetonitrile, while metal ion solutions were prepared in distilled water using their respective salts. Metal stock solutions (0.1 mM) were prepared by dissolving the salts in 100 mL of distilled water. Working solutions were then obtained by taking 5 mL of each metal stock solution and diluting it to 100 mL with distilled water. These working solutions of metal ions and the sensor JA were mixed in specific ratios, which are detailed in the results and discussion section, for subsequent UV–Vis and fluorescence measurements.

### 2.3 Excitation and emission wavelengths of optical sensor JA and Pb^2+^ complex

With excitation of optical sensor **JA** at *λ*ex = 370 nm, its fluorescence spectrum was recorded in a wide range of (240–800 nm). A weak fluorescence emission intensity was observed at *λ*em = 665 nm*.* Similar studies for the Pb(II) complex were also carried out in a wide range (220–800 nm) and the complex showed maximum emission at 665 nm, therefore all fluorimetric analysis were performed at these wavelengths of excitation and emission.

### 2.4 Water samples collection and spiking

Two distinct water samples were taken for this assessment. The first sample was obtained from the Analytical Chemistry Laboratory at University of Malakand, while the second sample was collected from the River Swat in Khyber Pakhtunkhwa, Pakistan. Samples preparation: Both water samples were subjected to a filtration process to remove any suspended particles. After filtration, the samples were stored in polyethylene bottles. These water samples were spiked with specific Pb^2+^ (5–30) *μ*M in the presence of **JA** (10 μM).

### 2.5 Theoretical calculation

The density functional theory (DFT) studies were achieved with the help of DMol3 simulation code [32], The geometrical and energetically analysis of the developed optical sensor **JA** were enhanced using Pardew Burke Enserch of functional (PBE) in the sphere general gradient approximation (GGA) [33]. The doubly numerically plus polarization (DNP)analysis [34], were employed to set all type of computational studies. Hessian data confirmed the unavailability of any associated imaginary temporary frequency showng that the relaxed structure is highly stable. The thermal smearing of parameters and cut-off were accustomed respectively to 0.005 au and 4.6 Å. Grime's scheme DFT-D2 empirical dispersion analysis was used for van der Waals intermolecular binding interactions determination [35], Convergence tolerance, of 10^−5^ Ha for the energy, 0.001H/Å for the force, and 0.005 Å for the movement were set at the time of geometrical relaxation process. The interaction energies (*E*_int_) for the complexes were investigated by using equation (1);

(1) $$E_{\mathrm{int}}=E_{\text{complex}}-\left(E_{JA}+E_{Pb\left(\text{II}\right)}\right)$$

Where, E_complex_, E_sensor,_ and E_Pb(II)_ are the total-electronic energies for Pb(II)-**JA** (complex), **JA,** and Pb(II), correspondingly.

### 2.6 Quantum yield calculation

Rhodamine 6G dye was used as a standard possessing quantum yield (Φ) 95 % for enumerating quantum yield of the sensor JA. For doing the experiments, Rhodamine 6G solution was prepared and its spectroscopic analysis (UV Visible and fluorescence) were performed. The quantum yield was determined with the help of given equation (2).

(2) $$\Phi_{JA}=\Phi_{R}\left(\frac{I_{JA}}{I_{R}}\right)\left(\frac{\eta_{JA}^{2}}{\eta_{R}^{2}}\right)\left(\frac{A_{R}}{A_{JA}}\right)$$

In this equation “JA” and “R” represent sensor and dye respectively. “I” stands for the integrated fluorescence intensity and “ŋ” corresponds to refractive index of solvent and “A” stands for absorbance.

## 3 Results and discussion

### 3.1 Synthesis and percentage yield of the sensor JA

The probe JA was synthesized using the reported protocol [36]. The percentage yield for the compound JA was calculated to be 75 %.

### 3.2 Optical properties of probe JA

In the present study we have studied the photo physical properties of optical sensor **JA** and its metal ions complexes. Due to the possessing of high photosensitivity and thermal stability of the synthesized compound, it was efficiently employed as optical sensors for trace-level metal ions detection in aqueous samples. The designed sensor possessed the best binding site (carbonyl functional group), with good binding ability. The sensor **JA** reflects a monodentate adept at binding to specific metal ions via the O atom of the carbonyl moiety. After analyte (metal ion) binding; the sensor JA for*m*ed complexes with metal ions, thereby produced changes in optical behavior that was easily detected by spectroscopic techniques.

### 3.3 UV–Visible study

**Fig. 2:**
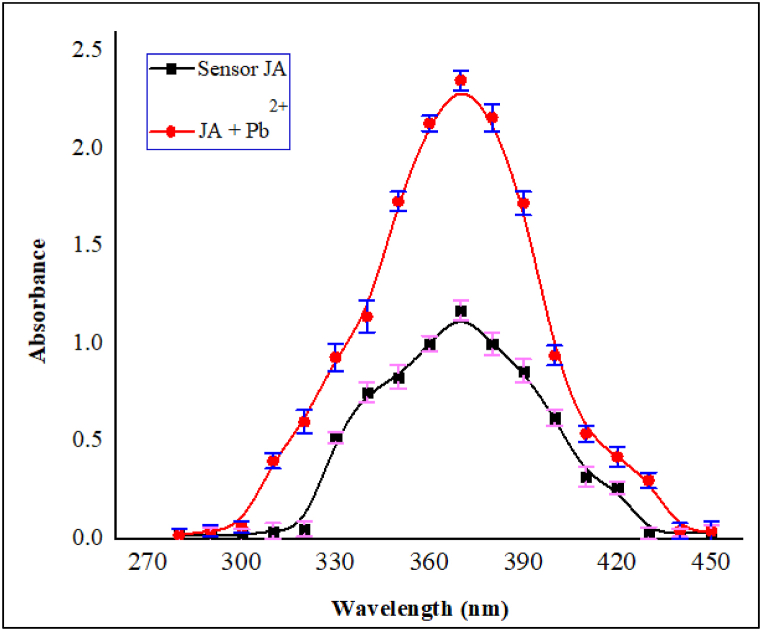
Absorption analysis of optical probe JA and JA-Pb^2+^ complex, using Pb^2+^ (20 μM) and sensor JA (10 μM) *λ*_max_ at 370 nm at room temperature.

The absorption peaks for sensor **JA** and sensor **JA**-Pb^2+^ complex were evaluated with the help of UV–Visible spectroscopy in acetonitrile as solvent system. For the determination of absorption maxima of the complex, Pb^2+^ (20 μM) aqueous solution was mixed with **JA** (10 μM) solution. The **JA** spectrum exhibited *λ*_max_ at 370 nm. Consequently, upon the addition of Pb^2+^ ion, an enhanced absorption occurred at *λ*_max_ of 370 nm, representing **JA**–Pb^2+^ ion complex formation. The absorption result is shown in Fig. 2. This region was regarded as sensor **JA** to metal charge transfer and the peak corresponded to the allowed electronic transition of n-ᴫ∗. It was mainly because of an overlap of Pb^2+^ orbital with sensor **JA,** which successively involved charge transfer and a sign of bonding interaction of **JA** with Pb^2+^ ion.

### 3.4 Fluorescence study

After the effective complex formation from absorption studies, fluorimetric studies were carried out to determine JA reactivity for Pb^2+^ ions.

#### 3.4.1 Initial fluorescence experiment

**Fig. 3:**
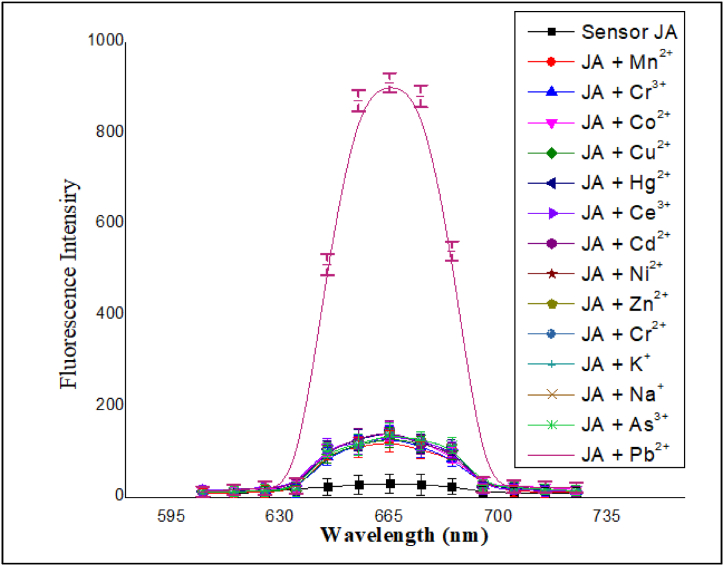
Initial fluorescence study of sensor **JA** at an optimized wavelength of excitation and emission 370 nm and 665 nm using (10 μM) and (200 μM) of sensor and Pb^2+^ respectively in a wide wavelength range of 220–800 nm.

This experiment was performed to determine the ability of the fluorescence sensor **JA** to detect various metal ions. Fluorescence-based methods are commonly used in analytical chemistry to detect and quantify specific analytes based on their ability to interact with fluorescent sensors. A set of different metal ions, including Mn^2+^, Cr^3+^, Pb^2+^, Co^2+^, Cu^2+^, Hg^2+^, Ce^3+^, Cd^2+^, and Ni^2+^, were

selected for testing. Each of these metal ions is known to have distinct chemical properties and may or may not form stable complexes with the fluorescence sensor **JA**. The metal ions were added to the **JA** solution at a concentration of 200 μM, while the **JA** solution itself was prepared at a concentration of 10 μM. This concentration difference allowed for a sufficient concentration gradient for potential complex formation. The metal ions were added to the **JA** solution in acetonitrile. Each metal ion (2 mL) was separately added to the **JA** (2 mL) solution allowing for the assessment of the sensor's response to each metal ion independently. Upon equating the spectra of sensor **JA** and sensor **JA**-metal ion complexes, the results showed that **JA** itself gave a weak emission intensity, whereas for **JA-**metal ion complexes, enhanced emission intensity took place and specifically for Pb^2+^, maximum enhancement was observed. The results are shown in Fig. 3. Therefore, **JA** was selected for the detection of Pb^2+^ ions only. The weak fluorescence intensity of **JA** can be attributed to an Intramolecular Charge Transfer (ICT) phenomenon [37]. In an ICT process, the electron density within a molecule is redistributed, leading to changes in its optical properties, such as fluorescence. When **JA** formed complexes with different metal ions, the fluorescence intensity enhanced. Furthermore, in the present study the enhancement in fluorescence intensity was due to complexation involving the formation of stable complex between **JA** and Pb(II) that positively influenced the fluorescence properties of the fluorophore in solution. This implies that the interaction enhanced the ability of the fluorophore to emit light when excited, resulting in an increase in fluorescence intensity [[38], [39], [40]]. Therefore, different parameters were optimized to enhance the sensitivity and selectivity of this optical sensor [41].

### 3.5 Optimization study

The optical response of metal ions toward JA can be affected by several parameters including pH, the concentration of probe and metal ions and the reaction time. Consequently, different fluorimetric studies were performed to obtain the optimum values for various parameters.

#### 3.5.1 Binding kinetics

A solution of **JA** at a concentration of 10 μM in acetonitrile was prepared. Also, the solution containing **JA** complexes with Pb^2+^ ions at a concentration of 20 μM in distilled water was prepared. The fluorescence spectra of these solutions were recorded over a time range of 5–35 min at an emission wavelength of 665 nm. From the recorded data, we observed that an instantaneous enhancement of fluorescence occurred after the binding of Pb^2+^ ions, indicating that the binding of Pb^2+^ ions to **JA** led to a rapid increase in fluorescence intensity. In contrast, when monitoring **JA** alone over a time range, there was no significant change in fluorescence intensity, suggesting that **JA** itself remained stable under the experimental conditions. It's common in fluorescence assays to monitor changes in fluorescence intensity over time to understand the kinetics of a reaction or interaction. In this case, the rapid enhancement of fluorescence when Pb^2+^ ions were introduced indicated a fast binding or interaction between **JA** and Pb^2+^ ions, leading to increased fluorescence [[42], [43]]. The obtained results are shown in Fig. S6.

#### 3.5.2 pH study

**Fig. 4:**
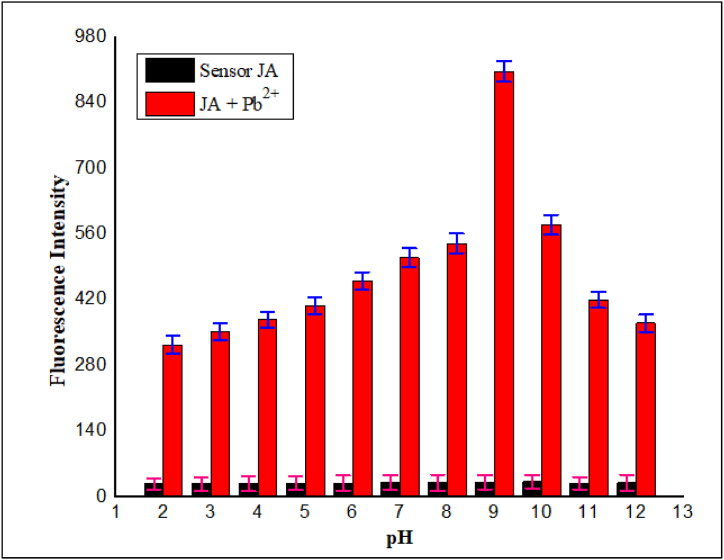
Effect of pH on JA and JA-Pb^2+^ complex, in pH range from 2 to 12, *λ*ex = 370 nm and *λ*em = 665 nm, result maximum fluorescence intensity at pH = 9.

When the pH was varied from 2 to 12, it was observed that the fluorescence intensity of **JA** did not significantly change within this pH range. This indicated that **JA** remained stable and its chemical structure was not affected by changes in pH. In contrast, when we investigated the behavior of the **JA**-Pb^2+^ complex under varying pH conditions interesting results were obtained. The fluorescence intensity of the **JA**-Pb^2+^ complex increased gradually upon increase in pH and maximum intensity was observed at pH 9 ^43^. However, at higher pH values (greater than 9), a gradual decrease in fluorescence intensity was observed as shown in Fig. 4. The reduction in fluorescence intensity at pH values above 9 can be attributed to a few factors. One possibility is the formation of hydroxide ions (-OH) in the alkaline environment. Additionally, the Pb^2+^ ions might form complexes with hydroxyl ions (Pb(OH)_2_), leading to a decrease in the concentration of available Pb^2+^ ions in solution. As a result, the complex formation between **JA** and Pb^2+^ ions became less favorable, causing a decrease in fluorescence. While on the other hand a relatively week fluorescence intensity was observed at acidic pH due to the protonation of the carbonyl oxygen due to which the complex was unstable thus quenched the fluorescence intensity. Based on these observations and the pH-dependent behavior of the **JA**-Pb^2+^ complex, we choose pH 9.0 as the optimal pH condition for our spectrofluorometric analyses. This pH level maximized the fluorescence intensity of the **JA**-Pb^2+^ complex, ensuring the best conditions for detecting and analyzing Pb^2+^ ions using **JA** as a sensor. This careful consideration of pH is crucial in fluorescence-based analytical techniques, as it can significantly influence the behavior and performance of fluorescent sensors and their interactions with the target analyte.

#### 3.5.3 Effect of JA concentration

**Fig. 5:**
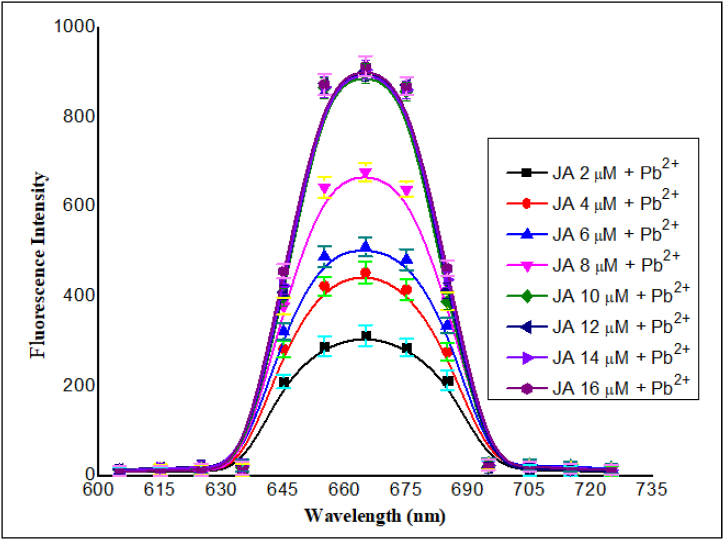
Effect of **JA** concentration (2–16 μM) and constant (20 μM) Pb^2+^ concentration at optimized wavelengths of excitation and emission.

The increasing **JA** concentration response on the fluorescence of **JA**-Pb^2+^ ion complex was recorded at constant (20 μM) Pb^2+^ concentration, while **JA** concentration was varied (in 2–16 μM) range. The fluorescence intensity of each sample was measured at a wavelength of emission λem = 665 nm upon exciting at λex = 370 nm (Fig. 5). The obtained results demonstrated that fluorescence emission intensity for the Pb^2+^ complex increased gradually upon an increase in **JA** concentration up to 10 μM. The fluorescence intensity of the complex remained constant at higher concentrations of **JA** due to the unavailability of free Pb^2+^ to bind with **JA.**

#### 3.5.4 Effect of concentration of Pb^2+^ ion

**Fig. 6:**
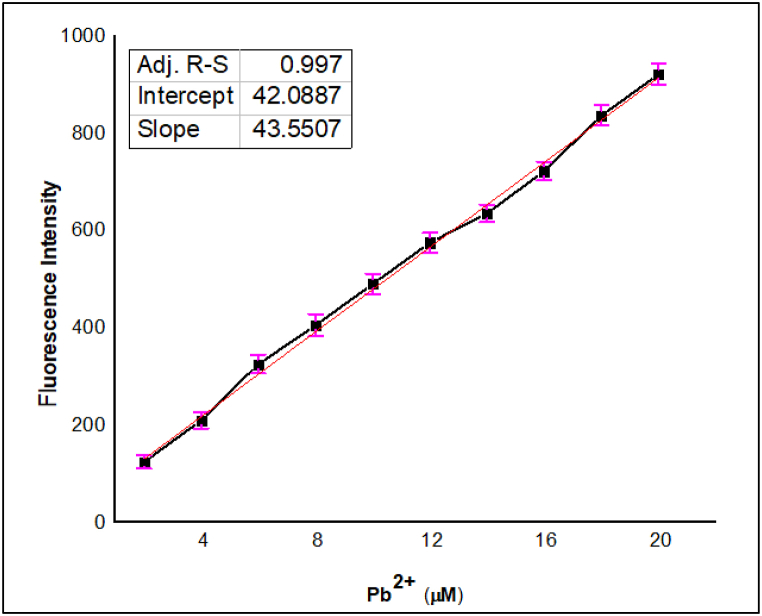
Effect of Pb^2+^ concentration (2–20 μM) range at constant JA (10 μM) indicating linear enhancement in fluorescence intensity up to 20 μM, *λ*ex = 370 nm and *λ*em = 665 nm.

A series of Pb^2+^ ion solutions were prepared in the concentration range of 2 μM–20 μM from a stock solution of a known Pb^2+^ concentration with distilled water. Similarly, the solution of optical sensor **JA** at a constant concentration of 10 μM. A fixed volume of the Pb^2+^ solution was added to the optical sensor solution. The fluorescence intensity for each of the different Pb^2+^ concentrations was recorded. A graph was plotted between fluorescence intensity against the concentration of Pb^2+^ ions. The results showed how the fluorescence intensity changed with increasing Pb^2+^ concentration. To check the control experiments, the fluorescence of the optical sensor was measured alone without Pb^2+^ ions, to account for any background fluorescence as shown in Fig. 6. At lower concentrations of Pb^2+^ ions (2–20 μM), the binding site was available on the sensors **JA**. With the increase in concentration of Pb^2+^ ions, more and more binding sites were occupied by Pb^2+^ ions, leading to an increase in the formation of the **JA**-Pb^2+^ complex. This increase in complex formation increased fluorescence intensity because the complex had different fluorescent properties compared to the free sensor **JA**. Similarly, in case of UV–Vis analysis: a linear increase in absorbance was observed upon increase in Pb(II) concertation in the range of (2–20) μM with constant probe concentration 10 μM. as shown in Fig. S3. Further increasing the Pb^2+^ ion concentration (beyond 20 μM) for both absorption and fluorescence studies didn't lead to a significant change in complex formation because there were no more available binding sites for additional Pb^2+^ ions [44]. The results obtained from fluorescence studies with higher concentration of Pb^2+^ is presented in Fig. S4.

#### 3.5.5 Job's plot analysis

**Fig. 7:**
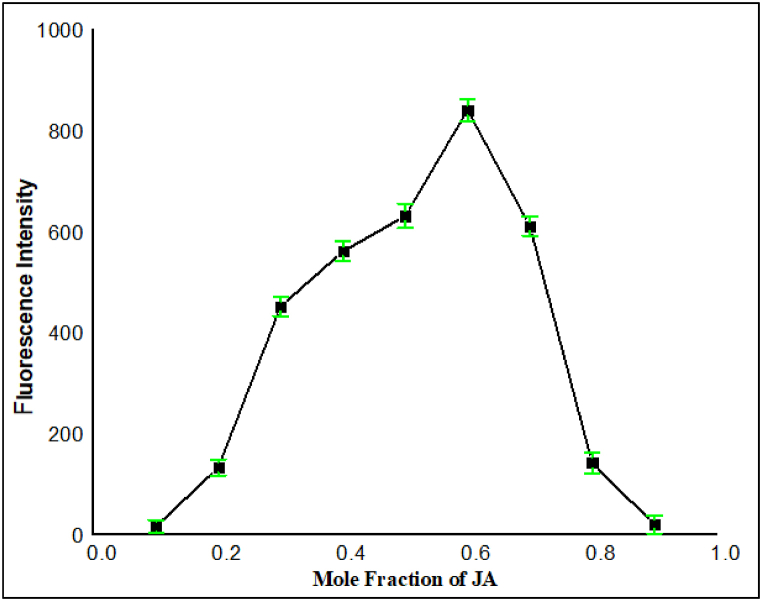
Job's plot analysis from fluorescence data at a variable mol ratio of **JA** and Pb^2+^ ion, demonstrating 2:1 complex formation between **JA** and Pb^2+^ ions.

In the current experiment, the overall concentration of **JA** and Pb^2+^ ions were retained at 10 μM (total concentration of the interacting species). While the molar ratio of **JA** to Pb^2+^ ions varied in the range of 0.1–0.9. For each molar ratio (0.1–0.9), we measured the fluorescence intensity of the system. The fluorescence emission intensity was drawn against the mole fraction of **JA** in the mixture. It was noted that the fluorescence was maximum at a molar fraction of 0.6 for **JA**. Thus indicated that at this specific composition (0.6 M fraction of **JA**), there was highest amount of complex formation between **JA** and Pb^2+^ ions [45]. Based on the maximum intensity point, inferred the stoichiometry of the complex formed. As the maximum intensity occurred at a molar fraction of 0.6, thus suggesting a 2:1 complex formation between **JA** and Pb^2+^ ions as shown in Fig. 7. This means that for every two molecules of **JA**, one molecule of Pb^2+^ ion was involved in the complex formation.

### 3.6 Proposed binding mechanism

The binding mechanism of current study is based on restriction of ICT phenomena. The complex formation could have caused a change in the electronic environment of the sensor, preventing charge transfer and resulting in increased fluorescence as shown in Fig. S7. Additionally, complex formation might also lead to a change in the conformation of the sensor, which affected its fluorescent properties. The proposed mechanism aligns with how complex formation can influence the fluorescent behavior of a molecule. By understanding the underlying change in molecular interactions and electronic properties, we can gain valuable insights into the sensor's response to the presence of Pb^2+^ ions [46]. Based on our current experimental condition the Pb(II) cannot get precipitated to hinder the fluorescence properties and the carbonyl group due to the possessing of lone pair of electron that can efficiently complex with Pb(II) ion thereby enhancing the fluorescence intensity. These proposed structures show the possible geometries and stabilities of the complexes formed between sensor JA and Pb(II).

### 3.7 Association constant determination

**Fig. 8:**
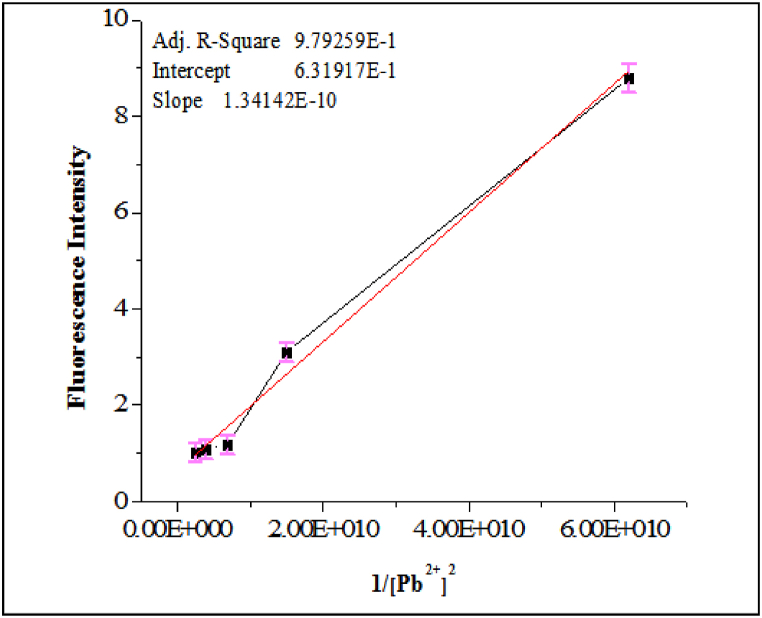
Benesi-Hilderbrand plot analysis for determining association constant, *λ*ex = 370 nm and *λ*em = 665 nm with Ka value of 3 × 10^6^ M^−2^ suggesting a relatively strong binding interaction between **JA** and Pb^2+^ ions.

For determining the association constant; the standard equation (4) was applied. In this investigation, sensor **JA** concentration was kept constant and Pb^2+^ concentration was varied. The fluorescence intensity was recorded for each Pb^2+^ concentration and is shown in Fig. 8. Ka was found to be 3 × 10^6^ M^−2^ for the complex^.^ This value represents the equilibrium constant for the formation of the JA and Pb^2+^ ions complex. A greater Ka showed a stronger binding interaction. Thus based on obtained data a Benesi–Hildebrand plot was drawn, which involved plotting the mutual of the change in fluorescence spectrum against the reciprocal of the Pb^2+^ concentration. The plot is typically linear, and its slope is equal to −1/Ka. The intercept on the y-axis is related to the fluorescence intensity of the receptor. The Benesi–Hildebrand plot allows you to visually assess the strength of the binding interaction between **JA** and Pb^2+^ ions. In the present case, we observe a linear relationship, and the slope of the plot would provide information about the association constant (Ka). A steeper slope (more negative) indicated a higher Ka and stronger binding. The Ka value of 3 × 10^6^ M^−2^ suggests a relatively strong binding interaction between **JA** and Pb^2+^ ions. This information is valuable for quantifying the strength of the complex formation and understanding the affinity of the sensor for Pb^2+^ ions. It also confirmed the results obtained from the Job's plot analysis, which indicated a 2:1 stoichiometry for the complex formation.

(3) F_max_-F_ₒ_/F-F_°_ = 1 + 1/K [Pb^2+^]^2^

### 3.8 Detection and quantification limits

In the current study, the detection limit (DL) was calculated to be 5 × 10^−7^ M, which means the method can reliably detect Pb^2+^ ions at concentrations as low as this value. The detection limit is often determined based on the signal-to-noise ratio and represents the smallest amount of analyte that produces a signal significantly above the background noise. Similarly, the limit of quantification (LOQ) was found 15 × 10^−7^ M, which means that we can accurately measure and quantify Pb^2+^ ions down to this concentration level with acceptable accuracy and precision. Photo physical properties of the sensor **JA** are given in Table 1. These values, DL and LOQ, provide important information about the performance of the analytical method. To quickly check whether Pb^2+^ ions are present or not, we can use the detection limit. If the concentration is below the DL, it suggests the analyte is likely not present at a significant level. When we need to measure the concentration of Pb^2+^ ions precisely, the LOQ is the relevant parameter. Concentrations above the LOQ can be accurately quantified [47]. The quantum yield calculated for the sensor JA was found to be 74 %.

**Table 1:** Photo physical properties of sensor **JA**.

*Λ*ex	*Λ*em	Association constant (M^−2^)	LOD (M)	LOQ (M)
370	665	3 × 10^6^	5 × 10^−7^	15 × 10^−7^

### 3.9 Interference study

**Fig. 9:**
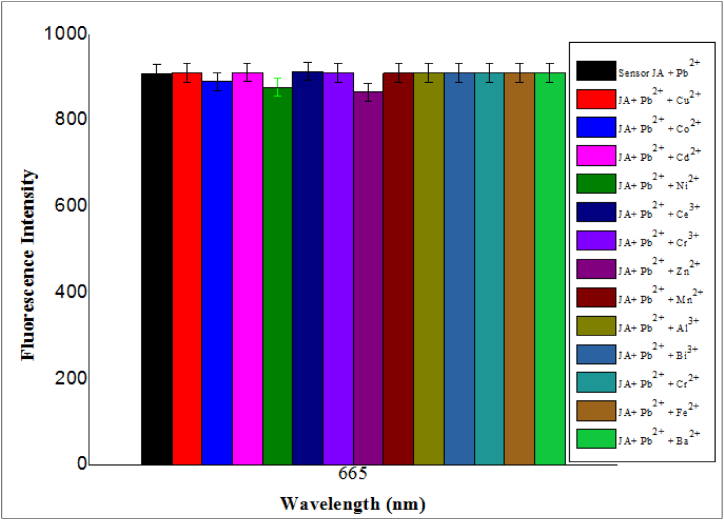
Interference study of optical sensor **JA**-Pb^2+^ complex, using optical sensor **JA** (10 μM), Pb^2+^ (20 μM), and other interfering metal ion (50 μM), *λ*ex = 370 nm and *λ*em = 665 nm, resulting no effect of these interfering metal ions.

We subjected sensor **JA** to several interfering metal ions, including mercury, copper, cobalt, manganese, cerium, chromium, cadmium, and nickel, under optimized conditions to check the selectivity of sensor **JA** for Pb^2+^. The concentration of sensor JA was kept constant at 10 μM, Pb^2+^ at 20 μM and the concentration of each interferer metal ion was 50 μM. The response of sensor J**A** to each of these metal ions is shown in Fig. 9. Each of these interfering metal ion solution and Pb^2+^ was added simultaneously to the sensor JA solution and its fluorescence response was measured. No significant change in fluorescence intensity was observed even in the presence of high concentration of interfering metal ion, thus proving the selective nature of **JA** for Pb^2+^. This selectivity is crucial for accurate and reliable detection of Pb^2+^ ions in environmental water samples, as it minimizes the chances of false positives interference from other metal ions [48]. This is particularly important in environmental monitoring and testing for heavy metal contamination, as Pb^2+^ ions can have detrimental effects on human health and the environment.

### 3.10 Environmental water analysis

**Fig. 10:**
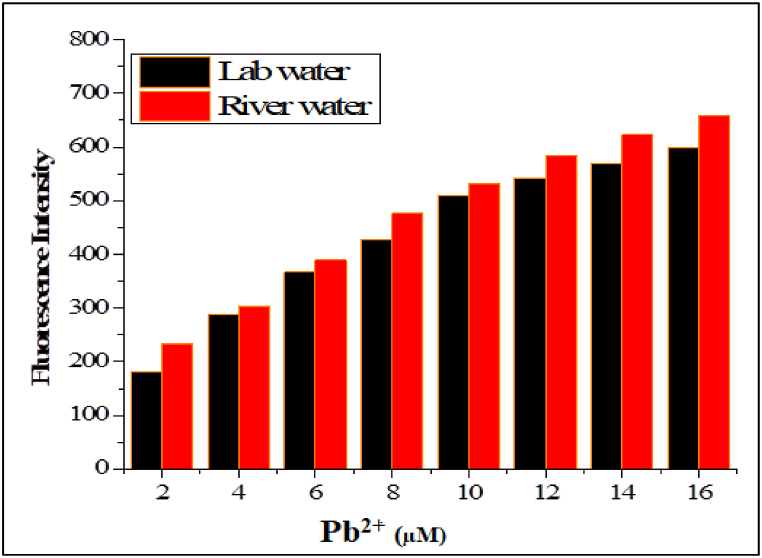
Real environmental sample analysis using sensor concentration JA (10 μM) and Pb^2+^ ion in range of 2–16 μM **at***λ*ex = 370 nm and *λ*em = 665 nm.

To check the real applications of the synthesized sensor **JA**, spiked natural water samples like tap water, and river water samples were analyzed. For spiking, Pb^2+^ ion solutions in a concentration range of 2–16 μM were prepared and added to the filtered natural water samples. These solutions likely served as a source of lead ions for the recovery tests. The results of these real water sample tests are presented in Fig. 10. This figure shows the response of the synthesized optical sensor **JA** to the added Pb^2+^ ions in both tap water and river water samples. These results indicate that the sensor responded to varying concentrations of Pb^2+^ linearly in these real-natural water samples thus demonstrating the sensitivity of the synthesized sensor **JA** and its reliability for the real-world recognition of Pb^2+^ ions in real samples. In other words, the sensor **JA** was able to effectively detect and quantify Pb^2+^ ions in environmental water samples, which is an important indication of its suitability for environmental monitoring and analysis [49]. The % recovery analyses for Pb^2+^ ion using sensor JA as an optical probe is given in Table 2. The results showed that sensor JA can efficiently be used for Pb^2+^ ion detection in environmental water samples.

**Table 2:** Recovery analysis for Pb^2+^ ion by employing sensor JA as optical sensor.

Type of sample	Amount of Pb^2+^ added (μM)	Amount of Pb^2+^ found (μM)	% Recovery	RSD
Tap water	5	4.8	96	±0.09
	10	9.7	97	±0.14
	15	14.6	97	±0.23
River water 1	10	9.3	93	±0.07
	20	19.3	96	±0.16
	30	29.0	96	±0.24
River water 2	10	9.5	95	±0.09
	20	19.3	96	±0.18
	30	29.2	97	±0.25

### 3.11 Reversibility experiment

The reversibility refers to the ability of a fluorescence-based analytical method/sensor to return to its original state after detecting an analyte or undergoing a reaction. In other words, a reversible sensor can be used multiple times to monitor changes in analyte concentration without being irreversibly altered by the analyte. To investigate the reversibility of the fluorescence response of sensor **JA** to Pb^2+^ ions, an experiment was conducted. In this experiment; sensor **JA** at a concentration of 10 μM. Pb^2+^ ions at a concentration of 20 μM and Ethylenediaminetetraacetic acid (EDTA) at a concentration of 20 μM, was added as a chelating agent. Fluorescence intensity was recorded at a specific wavelength (*λ*em = 665 nm). Here we have used EDTA as it is known for its ability to chelate metal ions and form stable complexes [50]. The results of the experiment indicated that by adding EDTA to the **JA**-Pb^2+^ ion complex, a decrease in fluorescence intensity was observed. This quenching suggested that the fluorescence enhancement previously observed in the presence of Pb^2+^ ions is reversible when EDTA is introduced, demonstrating the reversible nature of sensor **JA** response to Pb^2+^ ions. The results of this experiment are presented in Fig. S8, which presumably shows the fluorescence intensity changes at *λ*em = 665 nm as a function of EDTA addition to the **JA**-Pb^2+^ ion complex. The reversibility studies were performed six times by getting the same results. The obtained results showed the stability of fluorescence intensity of complex.

### 3.12 Computational detail

**Fig. 11:**
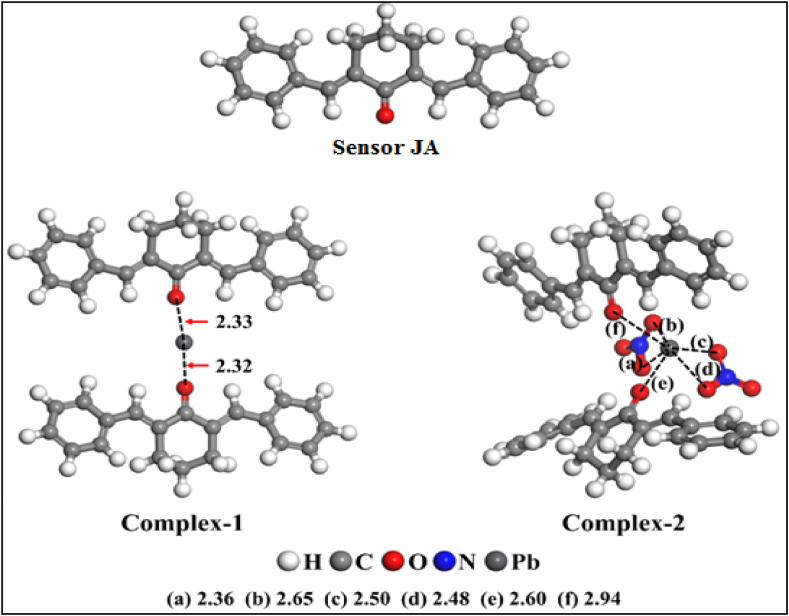
Enhanced geometries of optical sensor **JA** and Pb^2+^ complexes based on DFT results.

To gain insight into the process of removing Pb^2+^ ions from water using **JA**, it's essential to study the interactions between the sensor and metal ions at the atomic level. This involves examining how the electronic properties of both the sensor and the Pb^2+^ ions influence the binding process. The bonding interaction between the atoms of material is primarily driven as a result of the charged nature of the electrons. Understanding the electron distribution and behavior is crucial for comprehending chemical interactions and reactions. DFT is mentioned as an effective tool for studying the electronic properties of materials. It takes into account the positions and interactions of electrons, providing valuable insights into the behavior of materials at the atomic and electronic levels [51]. DFT is noted for considering the quantum mechanical nature of electrons. This means that it takes into account the wave-like properties of electrons, their energy levels, and their distribution in a material. The sensor has one carbonyl oxygen as a coordination site (act as monodentate) as shown in Fig. 11. The coordination of d-block elements including Pb^2+^ is either 4, but due to the inert pair effect, the 6S electron sometimes doesn't participate in complexation and their coordination number 2 can also be found. The bonding is strong, as the sensor is bulk, therefore, to get a complete understanding of the bonding strengths of the interaction sites i.e. Oxygen of the optical sensor JA and Pb^2+^, DFT simulations were carried out. Currently, the DFT models are a potent implement to calculate correctly the possible complex formation. In the current study, the DFT calculations were carried out with the help of DMOL3 code, Material Studio [52].

For the exchange-correlation purpose in these calculations, Perdew-Burke-Ernzerhof (PBE) design is used in the generalized gradient approximation (GGA). The DFT + D2 method (Grime's scheme) is used for a long-range dispersion correction in determining intermolecular binding. The double numerical plus polarization (DNP) basis set is employed for calculations, [53]. The Fermi-smearing is set to 0.005 Ha and a real space cutoff of 4.6 Å is utilized in the calculations. The geometries of all the systems are enhanced at convergence tolerance 10^−5^ Ha, 0.002 Ha/Å, and 0.005 Å for the energy, force, and displacement, respectively. Pb^2+^ ion in water was prepared by dissolving the Pb(NO_3_)_2_, and two types of complex i.e. Pb(sensor)_2_ and Pb(sensor)_2_(NO_3_)_2_ were considered.

**Fig. 12:**
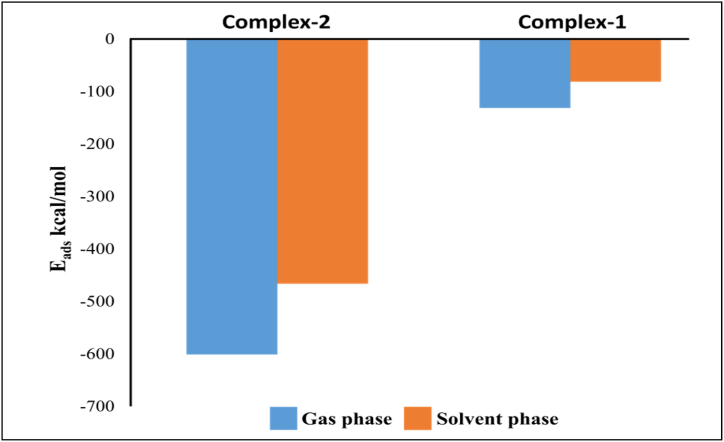
DFT results of optical sensor **JA** complex with Pb^2+^ indicating successful formation of sensor **JA** complex with Pb^2+^.

Therefore, a DFT study for two complexes was carried out as given in Fig. 11, Fig. 12. The binding energies are summarized in Table 2. Complex-1 was formed between Pb^2+^ and two carbonyl oxygen (C=O) sites of sensors having 2.33 and 2.32 Å distances whereas in the 2nd complex, Pb^2+^ ions bonded with two carbonyl oxygen (C=O) sites of two JA and four oxygen (O) of two NO_3_^−^ ions at a distance of 2.60 (e), 2.94 (f), 2.36 (a), 2.65 (b), 2.50 (c) and 1.48 Å (d). The interaction energies (E_int_) were calculated (Table-3) in gaseous and solvent phases for these two complexes are negative, proving energetically favorable complex formation. The E_int_ (gaseous state) for the 2nd complex (−601.06 kcal/mol) is relatively high as compared to complex-1 (−466.15 kcal/mol), which shows that complex-2 is more dynamically promising as compared to complex-1. The E_int_ order in the solvent (i-e water) state is identical to that of the gaseous state as the obtained values are lesser as compared to the gaseous state as a result of a larger dielectric constant value of water (78.5). Usually, with an increase in the dielectric constant of a medium, based on Coulomb law, the interaction of repulsive force among the charges decreases. The relative graphical illustration of both gaseous and solvent (water) states E_int_. Additionally, to check the interaction nature of Pb^2+^ with the optical sensor JA and NO_3_^−^ charge transfer differences (Δq_CT_) were analyzed with the help of Mulliken atomic charges determination. This indicates that after the interaction of Pb^2+^ with the sensor and NO_3_^−^, a decline was detected in the negative charge of O of sensor JA atoms and NO_3_^−^. Correspondingly, a decline in the positive charge of the Pb^2+^ ion was recorded. The optical performance of sensor **JA** as compared to existing sensors in the literature is presented in Table 4. Table 4 represent that sensitivity of current probe for Pb(II) ion detection in aqueous samples is higher as compared to most of similar published data in the literature [54].

**Table 3:** Binding energy of JA- Pb^2+^ complex in gas phase and solvent phase from DFT simulations.

Complex	Bond	Distance (Å)	E_int_ (Kcal/mol)		Δq_CT_
			**Gas phase**	**Solvent phase**	
1	A	2.33	−466.15	−81.23	−1.555
	B	2.32			−1.556
2	A	2.36			−1.208
	B	2.65	−601.06	−131.37	−1.102
	C	2.50			−1.143
	D	1.48			−1.165
	E	2.60			−0.981
	F	2.94			−0.956

**Table 4:** Significance of developed sensor **JA** over already existing sensors in literature.

Sensors	LOD (M)	LOQ (M)	Optical response	Analyte	Optimal pH	Testing media	Ref
CdTe quantum dots	7 × 10^−5^	21 × 10^−5^	turn-off	Pb^2+^	9	Environmental samples	[55]
MOF	8.4 × 10^−5^	25.2 × 10^−5^	turn-off	Pb^2+^, Hg^2+^	8	Aqueous samples	[56]
Gold nanoparticles	0.16 × 10^−6^	0.48 × 10^−6^	Fluorescence	Pb^2+^	NA	aqueous media	[57]
Carbazole based sensor	7.5 × 10^−7^	22.5 × 10^−7^	turn-on	Pb^2+^	9	Aqueous samples	[58]
trityl-picolinamide	5.7 × 10^−5^	17.1 × 10^−5^	Turn-on	Hg^2+^, Pb^2+^	8	Food Samples	[59]
Gold based sensor	5.9 × 10^−4^	17.7 × 10^−4^	Turn-off	Pb^2+^	9	Aqueous	[60]
Anthracene based probe	6.3 × 10^−6^	18.9 × 10^−6^	fluorescence off	Pb^2+^	7	Aqueous	[61]
Optical probes	8.6 × 10^−5^	25.8 × 10^−5^	turn-on	Ni^2+^, Pb^2+^	8	Aqueous	[62]
carbon based sensor	8.3 × 10^−6^	24.9 × 10^−6^	turn-off	Pb^2+^	9	Aqueous	[63]
Carbazole based probe	5.7 × 10^−6^	17.1 × 10^−6^	turn-on	Pb^2+^	8	Aqueous	[64]
**Sensor JA**	**5 × 10**^**−7**^	**15 × 10**^**−7**^	**turn-on**	**Pb**^**2+**^	**9**	**tape water**	**Current work**

## 4 Conclusion

In conclusion, our investigation centered on the synthesis of a fluorescent sensor, JA, designed for the detection of Pb^2+^ ions in water samples. A 2:1 ratio (sensor: Pb^2+^) binding stoichiometry was confirmed by job's methods and theoretical studies (DFT). Additionally, JA exhibited sharp sensitivity at pH 9, with a low detection limit (5 × 10^−7^ M). Notably, JA proved to be recyclable using EDTA as a chelating agent. This study underscores the potential of JA as an optical sensor with promising capabilities for specific metal ion (Pb^2+^) detection in environmental water samples, offering a vital tool for applications in environmental monitoring, industrial quality control, and other contexts where trace-level metal ion detection is essential. Based on our current experiments in future we have plan to synthesize similar curcumin based optical sensors and explore its bio imaging studies as well. Based on the obtained results, we anticipate that current work can be easily extended to tune the sensing performance of different heavy metals.

## CRediT authorship contribution statement

**Maria Sadia:** Writing – original draft, Supervision, Project administration, Methodology, Conceptualization. **Jehangir Khan:** Methodology, Investigation, Funding acquisition, Formal analysis, Data curation. **Rizwan Khan:** Methodology, Investigation, Funding acquisition, Formal analysis, Data curation. **Syed Wadood Ali Shah:** Methodology, Investigation, Formal analysis, Data curation, Conceptualization. **Adil Zada:** Methodology, Investigation, Funding acquisition, Formal analysis, Data curation. **Muhammad Zahoor:** Writing – original draft, Supervision, Project administration, Methodology, Conceptualization. **Riaz Ullah:** Writing – original draft, Resources, Methodology, Funding acquisition, Formal analysis, Conceptualization. **Essam A. Ali:** Writing – original draft, Methodology, Funding acquisition, Formal analysis, Data curation, Conceptualization.

## Consent to participate

N/A.

## Data availability

The authors declare that the data supporting the findings of this study are available within the paper and its Supplementary Information file. Should any raw data files be needed in another format they are available from the corresponding author upon reasonable request.

## Ethical approval

We declare that we have no human participants, human data, or human tissues.

## Consent for publication

N/A.

## Declaration of competing interest

The authors declare the following financial interests/personal relationships which may be considered as potential competing interests: Muhammad Zahoor reports was provided by University of Malakand. If there are other authors, they declare that they have no known competing financial interests or personal relationships that could have appeared to influence the work reported in this paper.
