# Supplementary material for: Trace level detection of Pb2+ ion using organic ligand as fluorescent-on probes in aqueous media
Source: Heliyon. 2024 Dec 12;11(1):e41125. doi: 10.1016/j.heliyon.2024.e41125 (PMC11719314; doi:10.1016/j.heliyon.2024.e41125)
Supplement: Multimedia component 1 [file mmc1.docx]

**Supplementary data**

**Trace level detection of Pb^2+^ ion using organic ligand as fluorescent-on probes in aqueous media**

Maria Sadia^1^ Jehangir Khan^1^, Rizwan Khan^2^, Syed Wadood Ali Shah^3^, Adil Zada^1^, Muhammad Zahoor*^4^, Riaz Ullah^5^, Essam A. Ali^6^

1. Department of Chemistry, University of Malakand, Chakdara, Lower Dir, Khyber Pakhtunkhwa, Pakistan: [mariasadia@gmail.com](mailto:mariasadia@gmail.com), jehangirchemist@gmail.com, [zadaadil899@gmail.com](mailto:zadaadil899@gmail.com)
2. Department of Electrical Engineering, Kwangwoon University Seoul, South Korea: [rizwanchemist700@gmail.com](mailto:rizwanchemist700@gmail.com)
3. Department of Pharmacy, University of Malakand, Chakdara, Lower Dir, Khyber Pakhtunkhwa, Pakistan: [pharmacistsyed@gmail.com](mailto:pharmacistsyed@gmail.com)
4. Department of Biochemistry, University of Malakand, Chakdara, Lower Dir, Khyber Pakhtunkhwa, Pakistan; [mohammadzahoorus@yahoo.com](mailto:mohammadzahoorus@yahoo.com)
5. Department of Pharmacognosy, College of Pharmacy, King Saud University, Riyadh, Saudi Arabia; [rullah@ksu.edu.sa](mailto:rullah@ksu.edu.sa)
6. Department of Pharmaceutical Chemistry, College of Pharmacy King Saud University Riyadh Saudi Arabia: [esali@ksu.edu.sa](mailto:esali@ksu.edu.sa)

* Corresponding author: [mohammadzahoorus@yahoo.com](mailto:mohammadzahoorus@yahoo.com)

1. **Synthesis of [2,6-di(*E*)-benzylidene) cyclohexane-1-one/(optical sensor JA)**

The optical sensor **JA** was synthesized by reacting benzaldehyde 12 mmol (1.44 g) and 6 mmol, (0.58 g) cyclohexanone in 12 mL ethanol, then 10 mL sodium hydroxide (10 %) aqueous solution was added. The compound was synthesized with the help of the reported protocol ^[30].[1-5]^. The mixture was followed by refluxing for 4 hours, and progress of the reaction was monitored using TLC. The reaction mixture was treated in ice and a mixture of HCl: water (30:70) after completion of reaction. As a result, the product obtained was yellow colored with a melting point of 162-164°C (yield 75%). The IR (KBr) spectrum (*νcm*^−1^): 2924-2836 (C-H asymmetric), 1611-1633 (C=C Ar), 1676 (C=O) 3034, (C-H, Ar). ^1^H-NMR spectrum of **JA**, δH (400 MHz, CDCl_3_), 7.81 (s, 2H), 7.27-51 (m, 10H, HAr), 2.92-2.95 (q, 4H, CH_2_) 1.82-1.73 (sq, 2H, CH_2_) ^[31] [12]^. The FTIR and NMR spectra are appendant as Figure S1 and Figure S2. Gao et-al (2011) synthesized and performed the 13C NMR analysis. The compound displayed characteristics peaks as given 13C{1H} NMR (75 MHz, CDCl3) δ 190.3, 137.0, 136.2, 136.0, 9 S 130.4, 128.4, 128.6, 28.5, 23.0 ppm. GC-MS m/z = 274 (M^+^) ^[13].^

1. **FTIR and ^1^H-NMR characterization**

The optical sensor **JA** was developed by catalyzing benzaldehyde and cyclohexanone in the presence of a base in ethanol and obtained in 75 % yield. With the help melting point (M.P) determination, the compound purity was checked. Fourier transform infrared measurements were employed for the structural interpretation of the developed sensor **JA**. FT-IR spectral data revealed different vibration modes as shown in Figure 1S. The moderate intensity peak at 1611-1633 cm^-1^ indicated aromatic conjugated C=C stretching vibrations. The IR band around 3034 cm^-1^ represented C-H aromatic stretching. The absorption peak around 1676 cm^-1^ was assigned to C=O stretching. The peaks around 1,561, 1,507, and 1,478 cm^-1^ were due to C=C bending vibrations of *α*, *β*-unsaturated carbonyl, and aromatic C=C, respectively. The ^1^H-NMR spectra (A, B, C D] of **JA** is shown in Figure 2S. The signals appeared in the range of 7.27-51 ppm, resulting in ten aromatic protons. The signal at 7.81 ppm was due to methylene proton. The multiplets at 22.92-2.95 ppm were due to the proton of the cyclohexanone ring ^[ 6-8]^.

1. **Solubility of lead in distilled water**

Lead nitrate is highly soluble in water, including distilled water. When lead nitrate is added to water, it dissociates into its constituent ions Both lead ions. Both lead ion and nitrate ion readily interact with water molecules, leading to the formation of a clear, colorless solution. The high solubility of lead nitrate in water is a characteristic property of many metal nitrates. ^[9-11]^

**Fig S1:** FT-IR spectrum of optical sensor **JA**


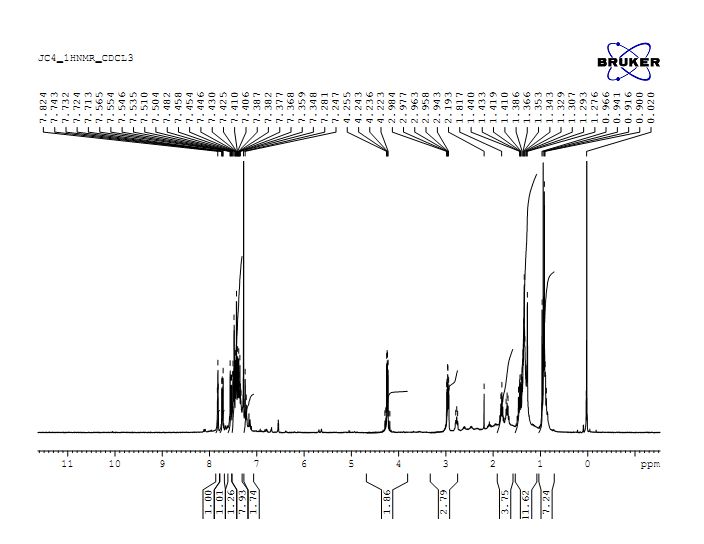


**
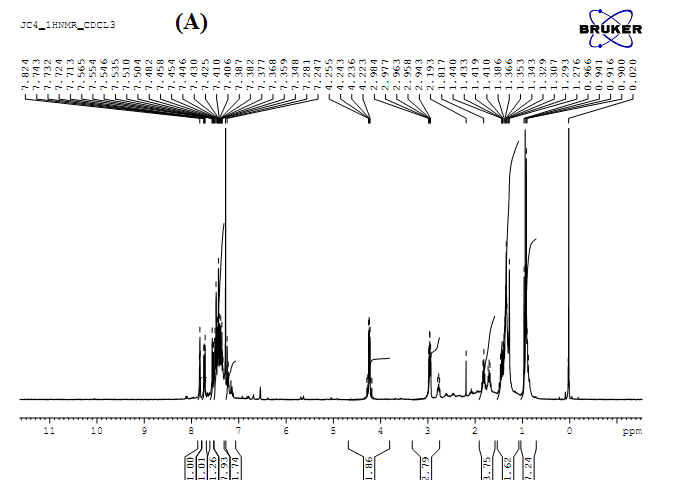
**

**
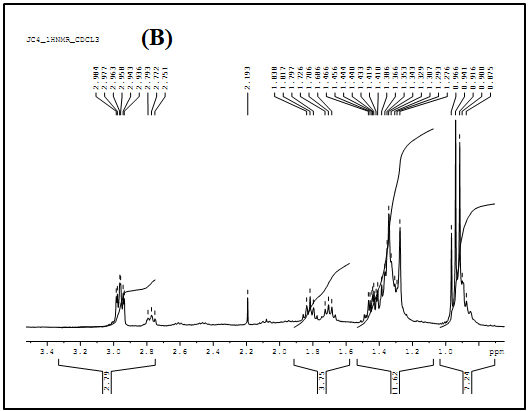
**

**
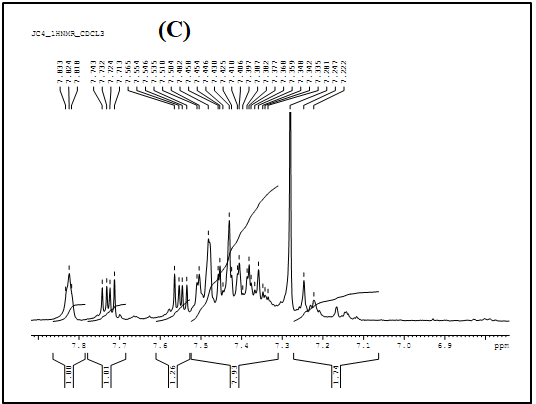
**

**
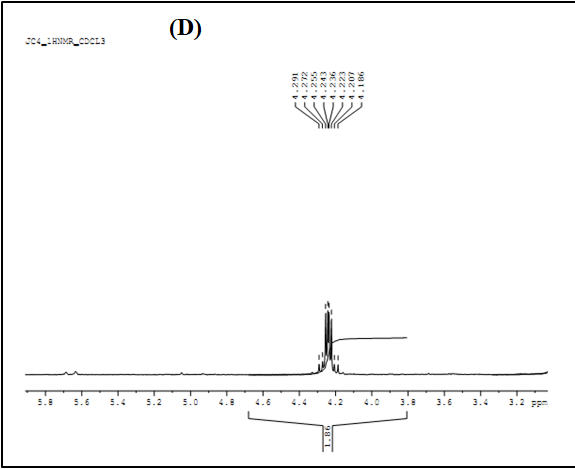
**

**Fig S2 (A, B, C, D):** ^1^H-NMR spectra of the optical sensor **JA,** demonstrating specific bands


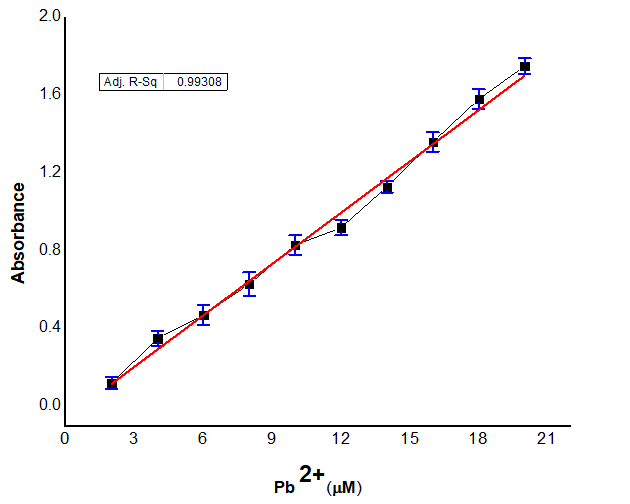


Figure S3. Effect of Pb^2+^ concentration (2-20 *µ*M) range at constant JA (10 *µ*M) from absorbance analysis at room temperature


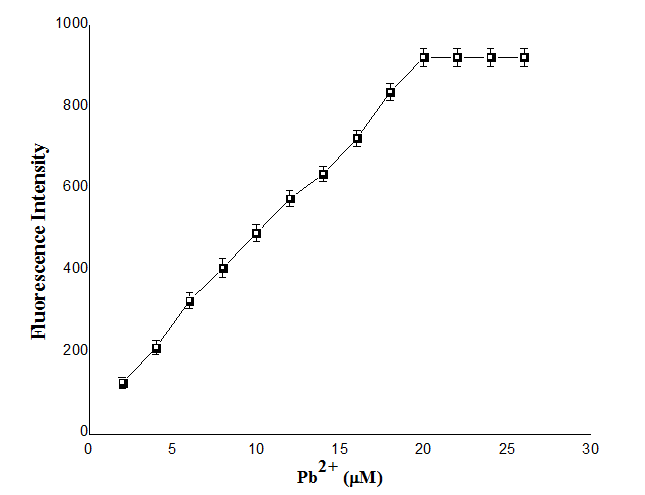


**Fig.S4**: Effect of increasing Pb^2+^ concentration (2-26 µM) at constant JA (10 *µ*M) on fluorescence intensity


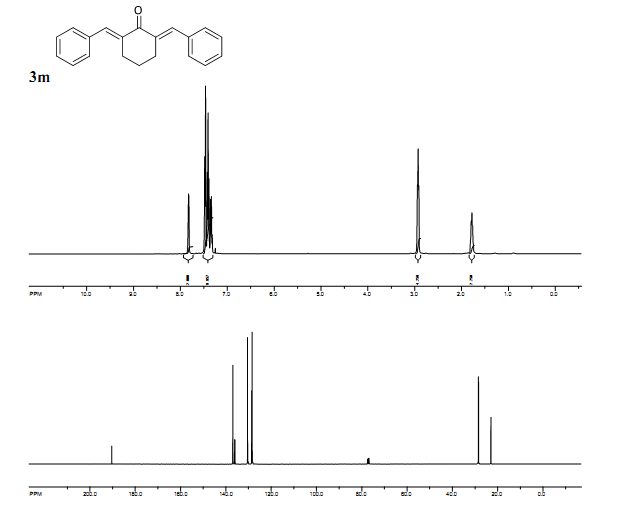


Figure S5. 13C{1H} NMR of sensor JA ^13^

#
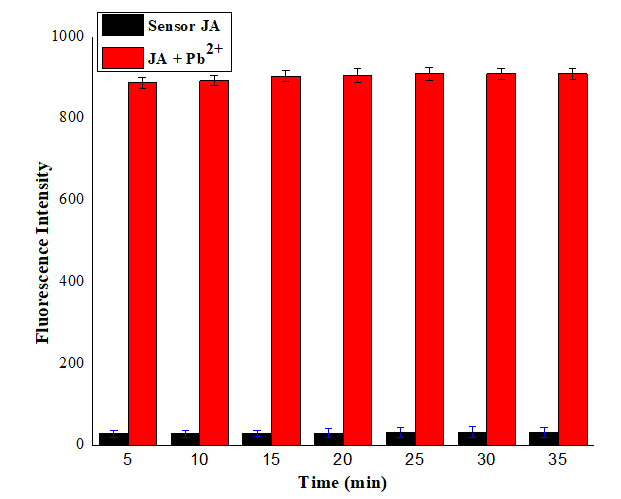


Fig. S6: Time study (5-35 min) of **JA** and **JA**-Pb^2+^ complex, at 665 nm wavelength of emission, at room temperature using JA concentration of 10 *μ*M in acetonitrile and Pb^2+^ 20 *μ*M in distilled water


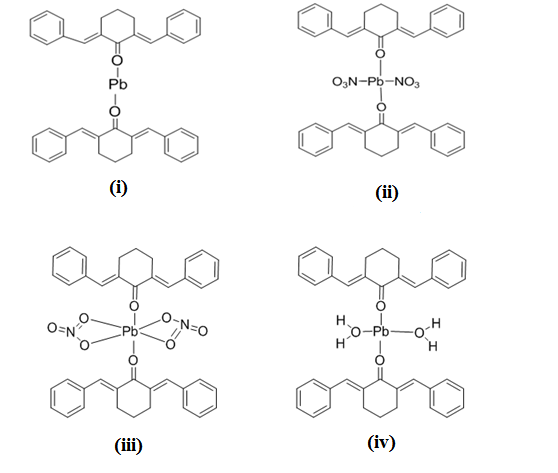


# Fig. S7. Proposed structures of JA-Pb^2+^ ion complex, based on binding ability of donor and receptor sites in sensor and lead ion


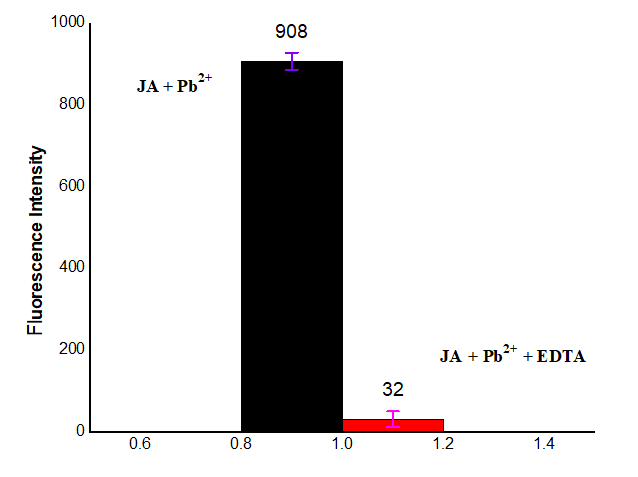


**Fig. S8.** Reversibility experiment using sensor **JA** (10 µM)**,** at *𝜆*em=665 nm, JA complex with Pb^2+^ ion (20 *µ*M) and EDTA (20 *µ*M), at fixed *λ*ex*=*370 nm

**References**

1. Lu, Z., Gao, Z., Song, H., Zhou, Y., Yuan, W., Wang, X., ... & Chang, C. (2023). Synthesis, Biological Evaluation and Action Mechanism Study of New Mitochondria‐Targeted Curcumin Derivative as Potential Antitumor Drugs. *Chemistry & Biodiversity*, *20*(7), e202300086.
2. Figueroa-DePaz, Y., Pérez-Villanueva, J., Soria-Arteche, O., Martínez-Otero, D., Gómez-Vidales, V., Ortiz-Frade, L., & Ruiz-Azuara, L. (2022). Casiopeinas of Third Generations: Synthesis, Characterization, Cytotoxic Activity and Structure–Activity Relationships of Mixed Chelate Compounds with Bioactive Secondary Ligands. *Molecules*, *27*(11), 3504.
3. Hassan, S. A. (2022). Synthesis and characterization of mixed ligand complexes from curcumin and new schiff base derived from isatin for some metallic ions and evaluation biological activities. *Research Journal of Pharmacy and Technology*, *15*(4), 1537-1542.
4. Ahsan, M. J., Choudhary, K., Ali, A., Ali, A., Azam, F., Almalki, A. H., ... & Salahuddin. (2022). Synthesis, DFT analyses, antiproliferative activity, and molecular docking studies of curcumin analogues. *Plants*, *11*(21), 2835.
5. Venkatas, J., Daniels, A., & Singh, M. (2022). The Potential of Curcumin-Capped Nanoparticle Synthesis in Cancer Therapy: A Green Synthesis Approach. *Nanomaterials*, *12*(18), 3201.
6. Ghaedrahmat, H., Masoomi, M. Y., & Zendehdel, M. (2023). Synthesize and characterization of ZIF-8/NaP zeolite composites as a stable acid-base catalyst for organic reactions. *Polyhedron*, *236*, 116372.
7. Li, Z. Y., Ding, L. L., Li, J. M., Xu, B. L., Yang, L., Bi, K. S., & Wang, Z. T. (2015). 1H-NMR and MS based metabolomics study of the intervention effect of curcumin on hyperlipidemia mice induced by high-fat diet. *Plos one*, *10*(3), e0120950.
8. Gören, A. C., Çıkrıkçı, S., Çergel, M., & Bilsel, G. (2009). Rapid quantitation of curcumin in turmeric via NMR and LC–tandem mass spectrometry. *Food Chemistry*, *113*(4), 1239-1242.
9. Kolthoff, I. M., Perlich, R. W., & Weiblen, D. (1942). The Solubility of Lead Sulfate and of Lead Oxalate in Various Media. *The Journal of Physical Chemistry*, *46*(5), 561-570.
10. Baltrusaitis, J., Chen, H., Rubasinghege, G., & Grassian, V. H. (2012). Heterogeneous atmospheric chemistry of lead oxide particles with nitrogen dioxide increases lead solubility: environmental and health implications. *Environmental science & technology*, *46*(23), 12806-12813.
11. Pierrard, J. C., Rimbault, J., & Aplincourt, M. (2002). Experimental study and modelling of lead solubility as a function of pH in mixtures of ground waters and cement waters. *Water Research*, *36*(4), 879-890.
12. Wu, Y., Hou, J., Liu, Y., Zhang, M., Tung, C. H., & Wang, Y. (2016). Chemoselective Claisen–Schmidt bis-substitutional condensation catalyzed by an alkoxy-bridged dinuclear Ti (IV) cluster. *Tetrahedron*, *72*(12), 1511-1516.
13. Gao, R., & Yi, C. S. (2011). Catalytic Formation of Silyl Enol Ethers and Its Applications for Aldol-Type Condensation and Aminomethylation Reactions. *ACS Catalysis*, *1*(5), 544-547.
